# The effect of patellar facet angle on patellofemoral alignment and arthritis progression in posterior-stabilized total knee arthroplasty without patellar resurfacing

**DOI:** 10.1186/s43019-020-00045-4

**Published:** 2020-06-09

**Authors:** Chang-Wan Kim, Chang-Rack Lee, Tae-Yung Huh

**Affiliations:** Department of Orthopedic Surgery, Inje University Busan Paik Hospital, 75, Bokji-ro, Busanjin-gu, Busan, 47392 South Korea

**Keywords:** Patella, Patellofemoral joint, Total knee arthroplasty, Treatment outcome

## Abstract

**Background:**

The purpose of this study was to evaluate the effect of patellar facet angle on pre- and postoperative patellofemoral alignment and the progress of arthritis of the patellofemoral joint in posterior-stabilized total knee arthroplasty (PS TKA) without patellar resurfacing.

**Methods:**

Patients who had a PS TKA for a varus osteoarthritic knee who were followed up for more than 2 years were included in this study. The radiologic and clinical outcomes were compared between 72 knees (group A) whose patellar facet angle was greater than 126° (> 126°) and 32 knees (group B) whose patellar facet angle was smaller than or equal to 126° (≤ 126°). For the radiologic assessment, the Kellgren-Lawrence grade, mechanical femorotibial angle, Insall-Salvati ratio, patellar tilt angle, patellar displacement and the osteosclerosis of the patellar ridge were evaluated. The range of motion (ROM) and patient-reported outcomes (the Knee Society knee score, the Knee Society function score, the Feller patellar score, and the Kujala patellofemoral score) were used for the clinical assessment.

**Results:**

The preoperative patellar tilt angle was 9.8° (standard deviation [SD] 5.5) and 14.6° (SD 4.1) in group A and group B, respectively, a significant difference (*p* < 0.001). Other preoperative radiologic parameters and preoperative patient-reported outcomes and ROM showed no significant difference between the two groups (all parameters (*p* > 0.05). At the last-follow-up, 22 knees (30.6%) showed progression of osteosclerosis of the patellar ridge in group A and 13 knees (40.6%) showed progression of osteosclerosis in group B (*p* = 0.371). The postoperative radiologic and clinical outcomes showed no significant difference between the two groups (all parameters, *p* > 0.05).

**Conclusions:**

Although a narrow patellar facet angle was related to an increase of lateral tilting of the patella, it showed no impact on the preoperative clinical assessment. The radiologic and clinical outcomes evaluated after the PS TKA showed no statistical difference according to the patellar shape. Although the patellar shape evaluated by the patellar facet angle can partially affect the preoperative patellofemoral alignment, this study result indicated insignificant clinical relevance of the patellar shape in the PS TKA.

## Background

Anterior knee pain is one of the main complaints among patients who have received a total knee arthroplasty (TKA) [1, 2]. The reported frequency of anterior knee pain after a TKA differs but between 6% and 25% of patients experience it after TKA with patellar retention [3]. Previous studies have suggested that a variety of surgery and prosthesis-related factors, including implant design, resurfacing of the patella, implant malpositioning, joint-line changes, and soft tissue impingement, are related to the anterior knee pain [4, 5]. To solve these problems, the design of the femoral and patellar component of the implant has been improved, making it more “patella-friendly”. In terms of the surgical technique, procedures, such as patellar resurfacing and lateral patellar facetectomy, are presently being tried [4–7]. Despite these efforts, however, anterior knee pain is still one of the main causes of dissatisfaction after TKA [8]. This might be attributed to factors other than the previously mentioned surgery and prosthesis-related factors. The patient-related factors related to anterior knee pain deserve particular attention because the anatomy of the patellofemoral joint, alignment and kinematics differ between patients [9, 10].

Some authors recently reported findings that the patellar shape is related to the radiologic and clinical outcomes after a patellar non-resurfacing TKA [9]. In that study, the patient group with a patellar facet angle smaller than 126° showed a larger lateral tilt angle of the patella after the arthroplasty and more frequent occurrence of progressive osteosclerosis of the patella, compared to the group whose patellar facet angle was greater than 126°. As the patellar shape differs among individuals, incongruity of the patellofemoral joint can occur if the patella is not resurfaced during TKA; this may be connected to the anterior knee pain [9, 10]. However, there are currently very few studies on this issue and the existing studies mainly investigated cruciate-retaining TKA (CR TKA). Because kinetics are different between posterior-stabilized TKA (PS TKA) and CR TKA [11, 12], studies of the effect of the patellar shape on outcomes after PS TKA are needed.

The purpose of this study was to evaluate the effect of patellar facet angle on pre- and postoperative patellofemoral alignment and the progress of arthritis of the patellofemoral joint in PS TKA without patellar resurfacing. We hypothesized that the patellar facet angle does not affect the radiologic and clinical outcomes after the PS TKA without patellar resurfacing.

## Methods

**Fig. 1:**
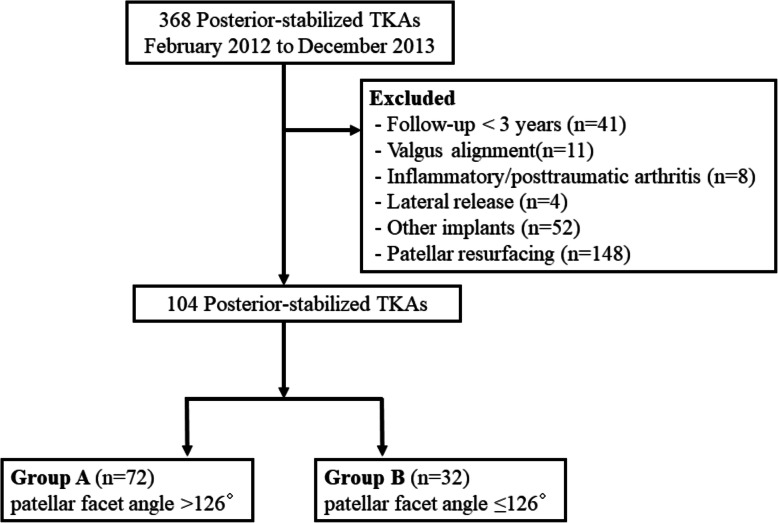
Patient flow chart

This study was approved by the Institutional Review Board of our institution (IRB No.20–0007). Between February 2012 and December 2013, TKAs were performed on 311 patients (368 knees) by a single surgeon in our institution. The patients who received a TKA for a varus osteoarthritic knee and were followed up for more than 2 years were included in this study. Patients were excluded from this study for the following reasons: the preoperative lower-extremity alignment was valgus, the TKA was due to inflammatory or posttraumatic arthritis, the patient had patellar resurfacing, developed complications, such as infection or periprosthetic fracture, and patients who had a lateral release due to the patellar maltracking during the operation. Since osteoarthritic knees with valgus alignment have different characteristics compared with varus osteoarthritic knees, such as contracture in the lateral soft tissues and hypoplasia of the lateral femoral condyle, they were excluded from this study [13–15]. The patients who received the TKA using implants other than the VEGA system (B. Braun, Aesculap, Tuttlingen, Germany) were also excluded from this study because the implant design can affect the evaluation of radiologic outcomes, such as patellofemoral alignment. Finally, 75 patients (104 knees) were enrolled in this study. Based on the receiver operating characteristic curve for the determination of the patellar facet angle cutoff value proposed in the study by Inoue et al. [9], patients were divided into two groups depending on whether their patellar facet angle was > 126° (group A) or ≤ 126° (group B) (Fig. 1). Group A had 72 knees and group B, 32 knees. The average follow-up duration after the surgery was 43.3 (standard deviation [SD] 6.6) months and 41.6 (SD 6.8) months for group A and group B, respectively. Patient demographics are described in Table 1.

**Table 1:** Sample characteristics

	Group A	Group B	*p* value
Knees (n)	72	32	
Sex (female/male)	65/7	30/2	0.718
Age at surgery	68.7 ± 7.0	69.4 ± 7.6	0.633
Follow-up period (months)	43.3 ± 6.6	41.6 ± 6.8	0.221
Body mass index (kg/m^2^)	25.2 ± 3.1	25.4 ± 2.5	0.804

### Surgical technique

The knee joint was exposed with the medial parapatellar approach after a midline skin incision. Bone resection of the distal femur, followed by the proximal tibia, then the anterior and posterior femur, was performed. The distal femur was resected at a valgus angle of 5–7° using an intramedullary alignment guide. The resection of the proximal tibia was performed to yield 3–5° of the posterior tibial slope using an intramedullary or extramedullary alignment guide. The medial 1/3 of the tibial tubercle, anterior tibial crest, and anterior tibial margin were used as a reference for the rotation of the tibial component. The femoral component was placed in 3–5° external rotation to the posterior condylar axis. Patellar resurfacing was selectively implemented in cases that had inflammatory arthritis or moderate to severe patellofemoral osteoarthritis.

### Evaluation

For the radiologic assessment, the Kellgren-Lawrence (KL) grade [16], hip-knee-ankle (HKA) angle, Insall-Salvati ratio, patellar facet angle, patellar tilt angle, patella displacement, and osteosclerosis of the patellar ridge were used. Standing knee anteroposterior (AP), lateral, Merchant, Rosenberg and standing whole leg AP radiographs were used. The radiologic parameters were measured from the preoperative radiographs and those taken at the last follow-up.

**Fig. 2:**
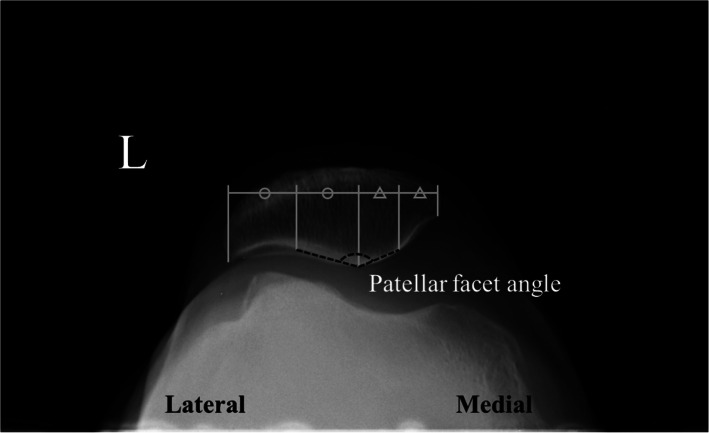
Measurement of patellar facet angle. **a** Patellar tilt angle, **b** patellar displacement

**Fig. 3:**
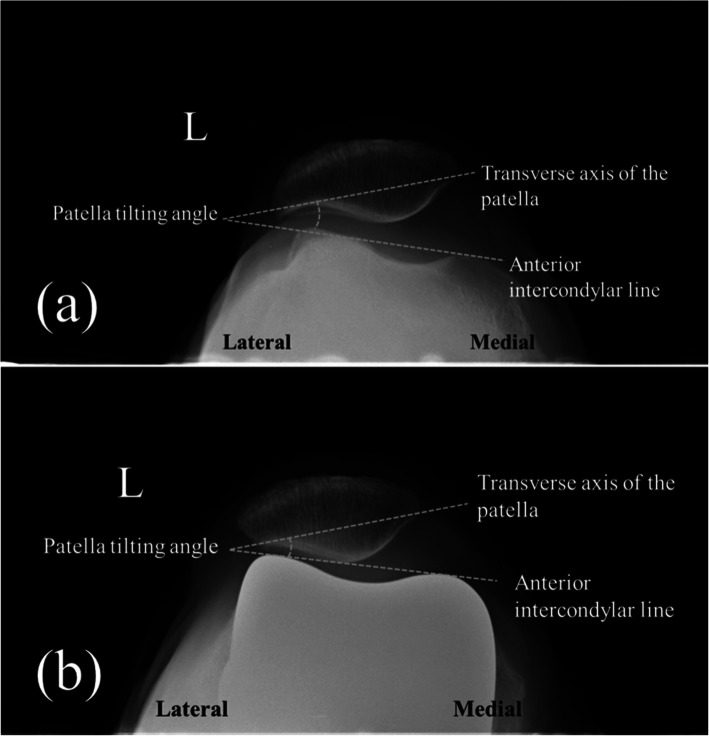
Measurements of patellar tilt angle and patellar displacement. **a** Preoperative, **b** postoperative

**Fig. 4:**
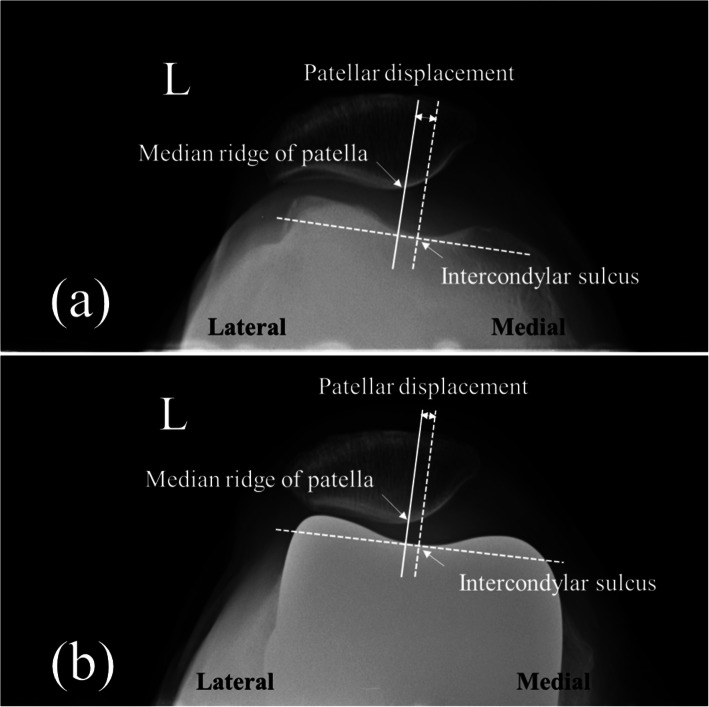
Measurements of patellar displacement. **a** Preoperative, **b** postoperative

**Fig. 5:**
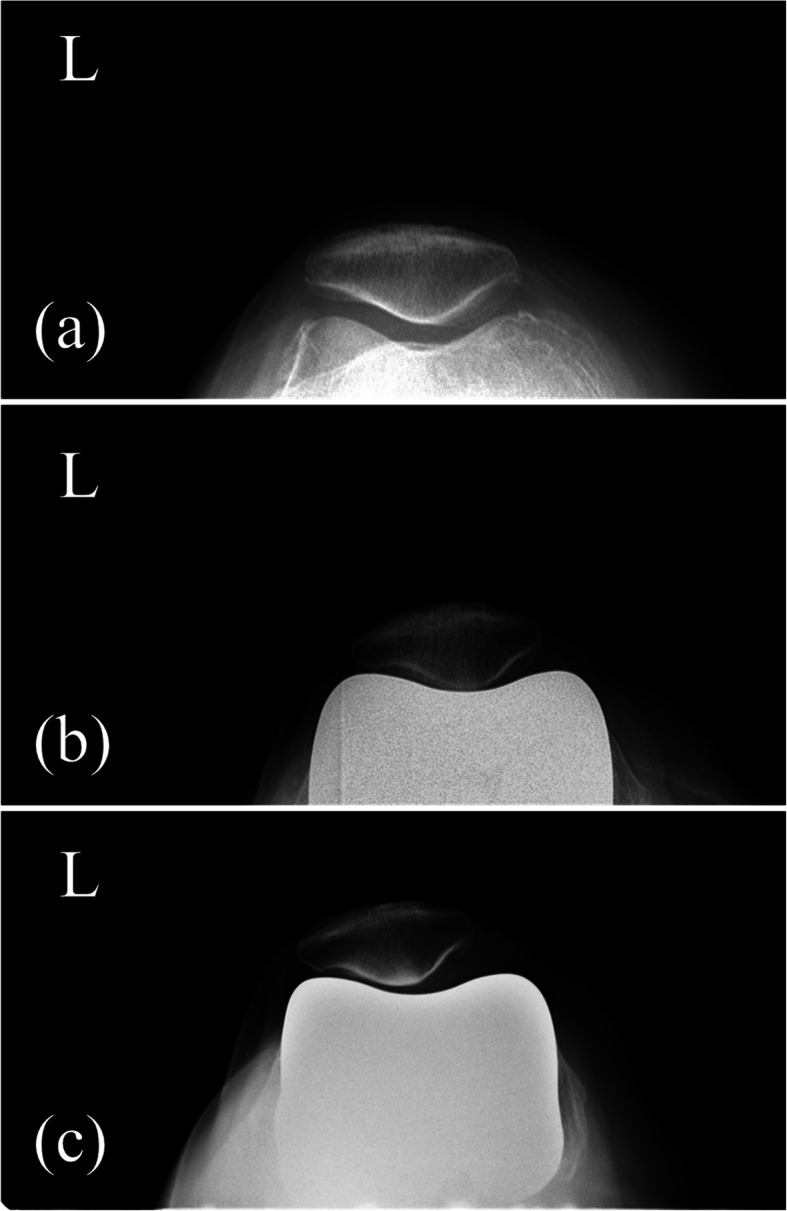
Progression of osteosclerosis. Progressive osteosclerosis was observed in the radiograph taken before the surgery (**a**), 1 year after the surgery (**b**), and 3 years after the surgery (**c**)

The KL grade was evaluated from the standing knee AP radiograph. The HKA angle is defined as an angle formed by the mechanical axis of the femur and the mechanical axis of the tibia on the weight-bearing whole leg AP radiograph. The HKA angle was expressed by the deviation from 180°. An angle smaller than 180° means varus alignment and greater than 180° means valgus alignment [17]. The Insall-Salvati ratio is defined as the ratio of the length of the patellar tendon to the maximum length of the patella [18, 19]. The patellar facet angle was evaluated on the Merchant view [9]. and is defined as the angle formed by a line that connects between the midpoint of the medial facet and central ridge and another line that connects between the midpoint of the lateral facet and central ridge (Fig. 2) For the evaluation of the patellofemoral alignment, the patellar tilt angle on the Merchant view and patellar displacement was used. The patellar tilt angle is defined as an angle formed by the anterior intercondylar line and transverse axis of the patella [20–22] (Fig. 3). The patellar tilt angle is expressed as a positive value when the transverse axis of the patella is tilted laterally from the anterior intercondylar line and as a negative value when it tilts medially. Patellar displacement is defined as the distance between the intercondylar sulcus and the median ridge of the patella [20–22]. The intercondylar sulcus is defined as the lowest point of the femur or femoral component on the anterior intercondylar line on the Merchant view. The median ridge of the patella is defined as the deepest point of the patella against the transverse axis of the patella (Fig. 4). The value is expressed as positive when the median ridge of the patella is located on the lateral side of the intercondylar sulcus and negative when it is on the medial side. The progression of osteosclerosis of the patellar ridge was evaluated in the Merchant view, where it was identified when the density of the patellar ridge at the last follow-up was greater than before the surgery (Fig. 5). To evaluate the patellar ridge, serial Merchant views were compared and the density of the patellar ridge in relation to the other areas of the patella were evaluated by two raters. Radiologic measurement was conducted using Picture Archiving Communication System software (Infinitt, Seoul, South Korea). This software has a minimum measurable angle change and length of 0.1° and 0.1 mm, respectively.

Clinical outcomes were evaluated by the range of motion (ROM) and patient-reported outcomes (PROs). The PROs included the Knee Society knee score [23], the Knee Society function score [23], the Feller patellar score [24], the Kujala patellofemoral score [25]. The PROs assessed before the surgery and the PROs obtained at the last follow-up observation were used in this study.

### Statistical analysis

The sample size was determined by referring to Inoue et al. [9], where it was determined that a total of 76 cases are needed to have a power of over 80% (≥) when the alpha level is set at 0.5. Our study included 104 cases. SPSS 25.0 (SPSS, Chicago, IL, USA) was used for the statistical analysis. An independent *t* test was used for the comparison of continuous variables between group A and group B. For the comparison of categorical variables, the chi-square test or Fisher’s exact test was used. The significance level was set at *p* < 0.05.

The test-to-test reliability of the radiologic measurements was evaluated. Each radiologic parameter was measured by two raters twice at 2-week intervals. The test-to-test reliability was evaluated using the intraclass correlation coefficient (ICC) for the continuous variables and kappa coefficient for the categorical variables. The ICCs of intra-observer reliability ranged between 0.86 and 0.92. The ICCs of inter-observer reliabilities ranged between 0.77 and 0.82. The kappa coefficient of intra-observer reliability ranged between 0.88 and 0.91 and the kappa coefficient of inter-observer reliabilities was ranged between 0.72 and 0.81. The values measured by the researcher with higher intra-observer reliability were used in this study.

## Results

Table 2 summarizes the preoperative radiologic and clinical assessments of group A and group B. The KL grade, HKA angle, Insall-Salviti ratio, and patellar displacement showed no significant differences between the two groups (all parameters. *p* > 0.05). The preoperative patellar tilt angle showed a significant between-group difference, where the patellar tilt angles of group A and B were 9.8° (SD 5.5) and 14.6° (SD 4.1), respectively (*p* < 0.001). The PROs and ROM evaluated before the surgery showed no significant differences between the two groups (all parameters *p* > 0.05).

**Table 2:** Preoperative radiologic and clinical assessment

	Group A	Group B	*p* value
**Radiologic**			
KL grade 3/4 (n)	20/52	10/22	0.815
HKA angle (°)	172.0 ± 5.6	171.4 ± 5.6	0.585
Insall-Salvati ratio	1.1 ± 0.2	1.1 ± 0.2	0.756
Patellar tilt angle (°)	9.8 ± 5.5	14.6 ± 4.1	< 0.001
Patellar displacement (mm)^a^	−1.0 ± 3.8	− 0.5 ± 3.3	0.431
**Clinical**			
KSKS	45.8 ± 12.7	46.9 ± 11.4	0.709
KSFS	36.3 ± 11.8	37.8 ± 10.9	0.547
Feller score	15.0 ± 5.7	14.3 ± 5.6	0.577
Kujala score	51.0 ± 17.7	50.6 ± 16.3	0.930
ROM (°)	118.2 ± 15.4	118.9 ± 15.0	0.858

*HKA* hip-knee-ankle, *KL* Kellgren-Lawrence, *KSFS* Knee Society function score, *KSKS* Knee Society knee score, *ROM* range of motion

^a^ A negative value means medial displacement and a positive value means lateral displacement

Table 3 summarizes the postoperative radiologic and clinical outcomes of the group. In the postoperative radiologic outcomes, the HKA angle of group A was 179.2° (SD 1.6) and the HKA angle of group B was 179.2° (SD 1.3) (*p* = 0.870). The postoperative patellar tilt angle and patellar shift were 9.0° (SD 6.4) and 0.9 mm (SD 3.7), respectively, in group A, while in group B, the postoperative patellar tilt angle and patellar shift were 11.9° (SD 10.2) and 0.3 mm (SD 3.6), respectively. There was no statistically significant difference (*p* = 0.147 and *p* = 0.511, respectively). In group A, 22 knees (30.6%) showed progression of osteosclerosis in the patellar ridge, while in group B, 13 knees (40.6%) showed progression. However, the difference was not significant. (*p* = 0.371). Postoperative clinical outcomes showed no significant differences between the two groups (all parameters, *p* > 0.05).

**Table 3:** Postoperative radiologic and clinical outcomes

	Group A	Group B	*p* value
**Radiologic**			
HKA angle (°)	179.2 ± 1.6	179.2 ± 1.3	0.870
Insall-Salvati ratio	1.1 ± 0.2	1.1 ± 0.1	0.866
Patellar tilt angle (°)	9.0 ± 6.4	11.9 ± 10.2	0.147
Patellar displacement (mm)^a^	0.9 ± 3.7	0.3 ± 3.6	0.511
Progression of osteosclerosis (n)^b^	22 (30.6%)	13 (40.6%)	0.371
**Clinical**			
KSKS	83.5 ± 12.1	86.3 ± 11.4	0.304
KSFS	81.3 ± 9.7	82.7 ± 8.7	0.505
Feller score	25.1 ± 4.1	25.5 ± 3.4	0.632
Kujala score	77.2 ± 10.7	75.6 ± 12.2	0.551
ROM (°)	127.9 ± 9.9	128.4 ± 9.4	0.844

*HKA* hip-knee-ankle, *KL* Kellgren-Lawrence, *KSFS* Knee Society function score, *KSKS* Knee Society knee score, *ROM* range of motion

^a^ A negative value means medial displacement and a positive value means lateral displacement

^b^ Evaluated at the patellar central ridge

## Discussion

The major finding of this study is that the patellar shape measured by the patellar facet angle does not affect the radiologic and clinical outcomes after a PS TKA although it can affect the patellofemoral alignment before the surgery.

Anterior knee pain is one of the main causes of dissatisfaction after TKA [1, 2, 8]. Previous studies have suggested a variety of causes including surgery-related factors, such as implant malpositioning or inaccurate surgical technique, and prosthesis-related factors, such as implant design [3–5]. However, patient-related factors should also be considered as a potential reason for the patellofemoral complication because the anatomy, alignment, and kinematics of the patellofemoral joint differ between the individuals [9, 10]. Some recent studies have reported the effect of TKA on patellar shape and clinical outcomes. Senioris et al. [26] followed up 30 patients for an average of 14 months after they had an uncemented mobile-bearing TKA and reported that there was a strong correlation between patellar shape and patellofemoral congruence, but these elements had no correlation with the clinical outcomes. However, Ait-Si-Selmi et al. [27] analyzed the correlation between clinical outcomes and preoperative patellar shape or postoperative patellar orientation of 144 patients, following them up for more than a year after performing a cemented PS TKA without patellar resurfacing. They reported that the pain and functional impairment after TKA were correlated with patellar shape. However, the aforementioned studies were all focused on patellar shape and clinical or functional outcomes. In contrast, another study evaluated patellar shape based on the patellar facet angle as well as the effect of the patellar shape on clinical and radiologic outcomes [9]. In their study, a patient group with a patellar facet angle ≤ 126° showed larger extents of lateral tilting after the TKA than those in which the angle was > 126°. The former group also showed more frequent development of progressive osteosclerosis of the patellar ridge at follow-up observation approximately 60 months postoperatively. The study included only patients who received CR TKA without patellar resurfacing. Different techniques of TKA include the CR type and PS type, depending on whether the posterior cruciate ligament (PCL) is preserved or not. Although several studies have reported that the two methods have similar clinical outcomes, it is not settled [28–30]. The two types of TKA do not have identical kinematics [11, 12]. Some authors have suggested that insufficiency of the PCL function affects the extent of the femoral rollback or paradoxical femoral roll forward in the CR TKA and that the changes in femoral rollback affect the patellofemoral contact load [31]. Furthermore, previous studies have noted that the PS TKAs that have a tibial post and femoral can show a higher frequency of complications, such as the patellar clunk syndrome, in the PS TKA [32–34]. Considering the difference of kinematics between CS TKA and PS TKA and the differences in the incidence of some patellar complications, research on the relationship between radiologic and clinical outcomes of the PS TKA and the patellar shape is necessary.

In this study, the group with a narrow patellar facet angle showed a greater preoperative patellar tilt angle than the control group. However, other radiologic parameters, including the Insall-Salvati ratio and patellar displacement, showed no significant differences between the two groups. Despite the difference in the patellar tilt angle, no significant between-group differences were observed in the preoperative clinical assessments. The findings of the preoperative radiologic and clinical assessments indicate that the patellar shape is one of the factors that can affect the preoperative patellofemoral alignment, despite the lack of clinical impact.

In the postoperative outcomes, there were no significant differences between the two groups in all radiologic and clinical parameters. Because it is difficult to accurately assess the postoperative patellofemoral alignment when implants with diverse designs are used, we included only patients with the same prosthesis in this study. Nevertheless, we could not confirm the correlation between the patellar shape and postoperative radiologic outcomes. The result implies that the patellofemoral alignment, including the patellar tilt angle, may be related to the soft tissue tension than to the patellar shape itself. In other words, this study result implies that the knees with a narrow patellar facet angle may have similar patellofemoral alignment with the knees whose patellar facet angle is > 126° as long as the soft tissue balancing and implant placement is appropriate.

Progressive osteosclerosis of the patellar ridge was observed in 30.6% in the group with a patellar facet angle > 126° and in 40.6% in the group with a patellar facet angle ≤ 126°, which was not a significant difference. This study result is not consistent with the results of Inoue et al. [9], who reported that a group with patellar facet angles ≤ 126° had more cases of progressive osteosclerosis. The inconsistency between the two studies can be attributed to the following reasons. First, this study is a short-term follow-up study where the average follow-up duration was approximately 40 months. The duration could have been too short to assess the progression of osteosclerosis. Second, differences in the implant used may have had an effect. In this study, the same implant type was used in all cases. Finally, as previously mentioned, the effects of the patellar shape on the alignment or kinematics of the patellofemoral joint can be trivial when the soft tissue balancing and implant placement is appropriate. However, the precise reason cannot be clarified at present and mid- and long-term follow-up observation will be necessary in the future.

This study has several limitations. First, it is a retrospective study with a small number of cases. Second, it is a short-term follow-up study. As a result, radiologic parameters, such as osteosclerosis, might have changed at the time of a mid- or long-term follow-up study. Third, all cases included in this study received the TKA using a modified gap technique by an experienced surgeon. The operator’s experience or surgical technique can be one of the elements that have affected the result. Fourth, since only one type of implant was used in this study, selection bias should be considered when interpreting the results. Fifth, the progression of osteosclerosis of the patellar ridge was not quantified; however, it was evaluated by two raters to increase the reliability. Sixth, in this study, patients who received bilateral TKAs were included. However, since the number of these patients was too small, this study could not evaluate patellar facet angle effect on the pre- or postoperative radiologic outcomes in the bilateral TKAs. Finally, in this study, the effects of the patellar facet angle on the postoperative radiologic outcomes were evaluated by comparing two groups using the independent *t* test and chi-square or Fisher’s exact test. The comparison showed that there was no significant difference between the two groups. However, this result does not mean that the radiologic results between the two groups were statistically identical since a non-inferiority test was not conducted in this study. Care should, therefore, be taken when analyzing the study results.

## Conclusions

Although a narrow patellar facet angle was related to an increase of lateral tilting of the patella, it showed no impact on the preoperative clinical assessments. The radiologic and clinical outcomes evaluated after the PS TKA showed no difference according to the patellar shape. Although the patellar shape evaluated by the patellar facet angle can partially affect the preoperative patellofemoral alignment, this study result indicated insignificant clinical relevance of the patellar shape in the PS TKA.

## Data Availability

Not applicable.

## Abbreviations

AP: Anteroposterior
CR TKA: Cruciate-retaining total knee arthroplasty
HKA: Hip-knee-ankle
ICC: Intraclass correlation coefficient
KL: Kellgren-Lawrence
PCL: Posterior cruciate ligament
PRO: Patient-reported outcome
PS TKA: Posterior-stabilized total knee arthroplasty
ROM: Range of motion
SD: Standard deviation
TKA: Total knee arthroplasty
